# Molecular Identification of Multiple Antibiotic Resistant Fish Pathogenic *Enterococcus faecalis* and their Control by Medicinal Herbs

**DOI:** 10.1038/s41598-017-03673-1

**Published:** 2017-06-16

**Authors:** Muntasir Rahman, Md. Mahbubur Rahman, Suzan Chandra Deb, Md. Shahanoor Alam, Md. Jahangir Alam, Md. Tofazzal Islam

**Affiliations:** 1 Department of Biotechnology, Bangabandhu Sheikh Mujibur Rahman Agricultural University, Gazipur, 1706 Bangladesh; 2 Department of Genetic Engineering and Biotechnology, Shahjalal University of Science and Technology, Sylhet, 3114 Bangladesh; 3 Department of Genetics and Fish Breeding, Bangabandhu Sheikh Mujibur Rahman Agricultural University, Gazipur, 1706 Bangladesh; 4 Department of Fisheries Biology and Aquatic Environment, Bangabandhu Sheikh Mujibur Rahman Agricultural University, Gazipur, 1706 Bangladesh

## Abstract

The opportunistic fish pathogen, *Enterococcus faecalis* has been reported to cause mass mortality in several fish species in different countries. The objectives of this study were to (i) identify *E*. *faecalis* from the diseased fishes through molecular techniques; (ii) assess the antibiotic susceptibility profile of *E*. *faecalis* isolates; and (iii) control disease in tilapia fish by treatment with medicinal plant extracts. A total of 48 isolates were phenotypically identified as *Enterococcus* species from tilapia, stinging catfish and walking catfish cultivated in several fish farms in Gazipur. Ten randomly selected isolates were identified as *E.* *faecalis* by 16S rRNA gene sequencing. Artificial infection revealed that most of the isolates caused moderate to high mortality in fishes with characteristic disease symptoms. These isolates exhibited resistance to multiple antibiotics *in vitro*. Bioassay revealed that organic extracts of *Tamarindus indica* and *Emblica officinalis* leaves, *Allium sativum* bulb, and *Syzygium aromaticum* bud inhibited the growth of *E*. *faecalis*. Methanol extracts of *A*. *sativum* and methanol and acetone extracts of *S*. *aromaticum* significantly reduced the mortality of fish artificially infected with *E*. *faecalis* as both preventive and therapeutic agents. This is the first report on molecular identification, and herbal control of fish pathogenic *E*. *faecalis* in Bangladesh.

## Introduction

Bacteria are the leading causative agents of diseases in freshwater fishes all over the world^1^. *Aeromonas*, *Edwardsiella*, *Pseudomonas*, *Flavobacterium*, *Vibrio* and *Streptococcus* are major genera of fish pathogens causing diseases in different tropical freshwater fishes^2^. In recent years, some opportunistic bacterial fish pathogens have been identified as the causal agents for severe outbreaks in aquaculture facilities. Among them, *Enterococcus* sp. has emerged as one of the important fish pathogens, which severely impacts aquaculture practices worldwide^3^. The incidence of fish diseases caused by *Enterococcus* sp. was first reported in Yellow tail (*Seriola quinqueradiata*) in Japan^4^ and then in Turbot (*Scophthalmus maximus*)^5^ and tilapia(*Oreochromis niloticus*)^6^. *E*. *faecalis* has been reported as a pathogen causing streptococcal infection in tilapia in lakes of Egypt, and Thailand^7–9^. In Bangladesh, *Enterococcus* sp. is often isolated from both healthy and infected fish^10–12^. Bangladesh is ranked fifth among the inland aquaculture producing countries in the world^13^. However, no studies have so far been conducted on the pathological involvement of *E*. *faecalis* in aquaculture in Bangladesh.

Antibiotic resistance is a great concern in the management of bacterial diseases worldwide. *Enterococcus* shows resistance against a wide range of antibiotics^14^. However, no information is available on the antibiotic susceptibility profile of *E*. *faecalis* isolated from the diseased fish in Bangladesh. As antibiotic resistance is a growing concern for management of bacterial diseases, alternative disease management strategies are needed. Extracts of medicinal plants exhibit antibacterial activities against human, plant, and fish pathogens^15^. Bangladesh is rich in diverse traditional medicinal plants^16^. However, no study has so far been conducted on management of fish diseases caused by *E*. *faecalis* using medicinal plant extracts from Bangladesh. Therefore, the objectives of this study were to (i) identify *E*. *faecalis* from the diseased fishes through molecular techniques; (ii) assess the antibiotic susceptibility profile in isolated fish pathogenic *E*. *faecalis*; and (iii) control of disease in *O*. *niloticus* fish by treatment with medicinal plant extracts.

## Results

### Isolation and Identification of Bacteria from Infected Fish

A total of 48 bacterial strains were isolated from the infected tilapia and catfish. Morphological studies revealed that all of these isolates gave small to medium sized, circular, smooth and raised colonies on KF Streptococcal agar plates. All of them formed dark red colored colonies on KF Streptococcal agar media but formed creamy transparent colored colonies when grown on nutrient agar media. All isolates were Gram positive, cocci, non-motile, catalase and oxidase negative, D-glucose fermentative, and methyl red, Voges-Proskauer and indole positive. They grew in the presence of 6.5% NaCl, 40% bile salts, 0.1% methylene blue milk at pH 9.6 and at 10 °C and 45 °C (Table 1). Based on the morphological, physiological and biochemical characteristics, all the isolates were tentatively identified as *Enterococcus* species. Among them, 10 isolates were randomly selected for further molecular, pathological, antibiotic susceptibility and herbal disease control studies.

**Table 1:** Morphological, physiological and biochemical characters of 48 *E*. *faecalis* isolates collected from infected fishes in Bangladesh.

Test Type	Tests	Characteristics
Colony characters	Size	M
	Type	R
	Color	Dark red
	Shape	C
Morphological character	Shape	Cocci
Physiological characters	Motility	−
	Growth at 10 °C	+
	Growth at 45 °C	+
	Growth in 40% bile salt	+
	Growth in 0.1% methylene blue milk at pH 9.6	+
	Growth in 6.5% NaCl	+
Biochemical characters	Gram’s staining	+
	Catalase	−
	Oxidase	−
	Oxidative-Fermentative	F
	Methyl Red	+
	Voges-Proskauer	+
	Indole	+

M: medium; R: round; C = convex; F: Fermentative; +: positive; − : negative.

The 16S rRNA gene sequence data of the ten selected isolates exhibited 99.62 to 99.93% homology with *E*. *faecalis* strain ATCC 19433. The sequences of the isolates have been deposited to the NCBI GenBank. The source of isolation, sequence homology with *E*. *faecalis* strain ATCC 19433 and NCBI GenBank accession number are presented in Supplementary Table S1.

### Molecular Detection of *E*. *faecalis* by Specific Primer

**Figure 1:**
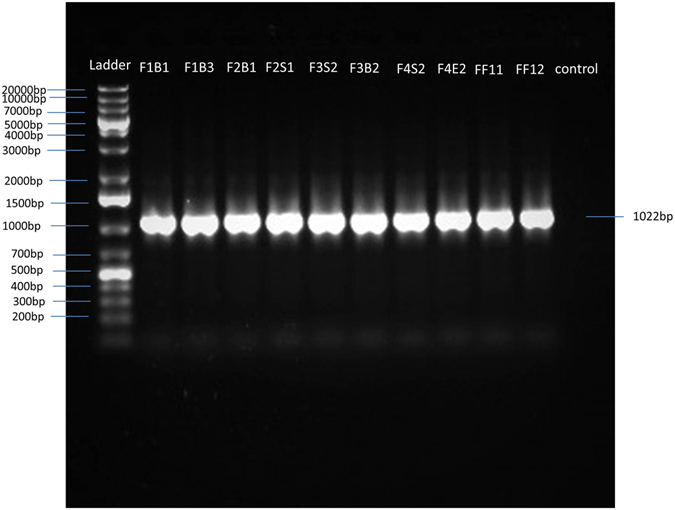
PCR amplification of *E*. *faecalis* isolates with specific forward (Efac F1) and universal reverse (1492R) primer. Ladder, 1 kb plus DNA ladder.

Amplification of a 1022 bp *E*. *faecalis* specific PCR product was obtained in the present study by combination of the *E*. *faecalis* specific forward primer EfacF1 and a universal reverse primer 1492R (Fig. 1). The EfacF1 and universal 1492R primer set amplified *E*. *faecalis* isolates only.

### *In vivo* Infection Model of the Isolated *E*. *faecalis*

**Figure 2:**
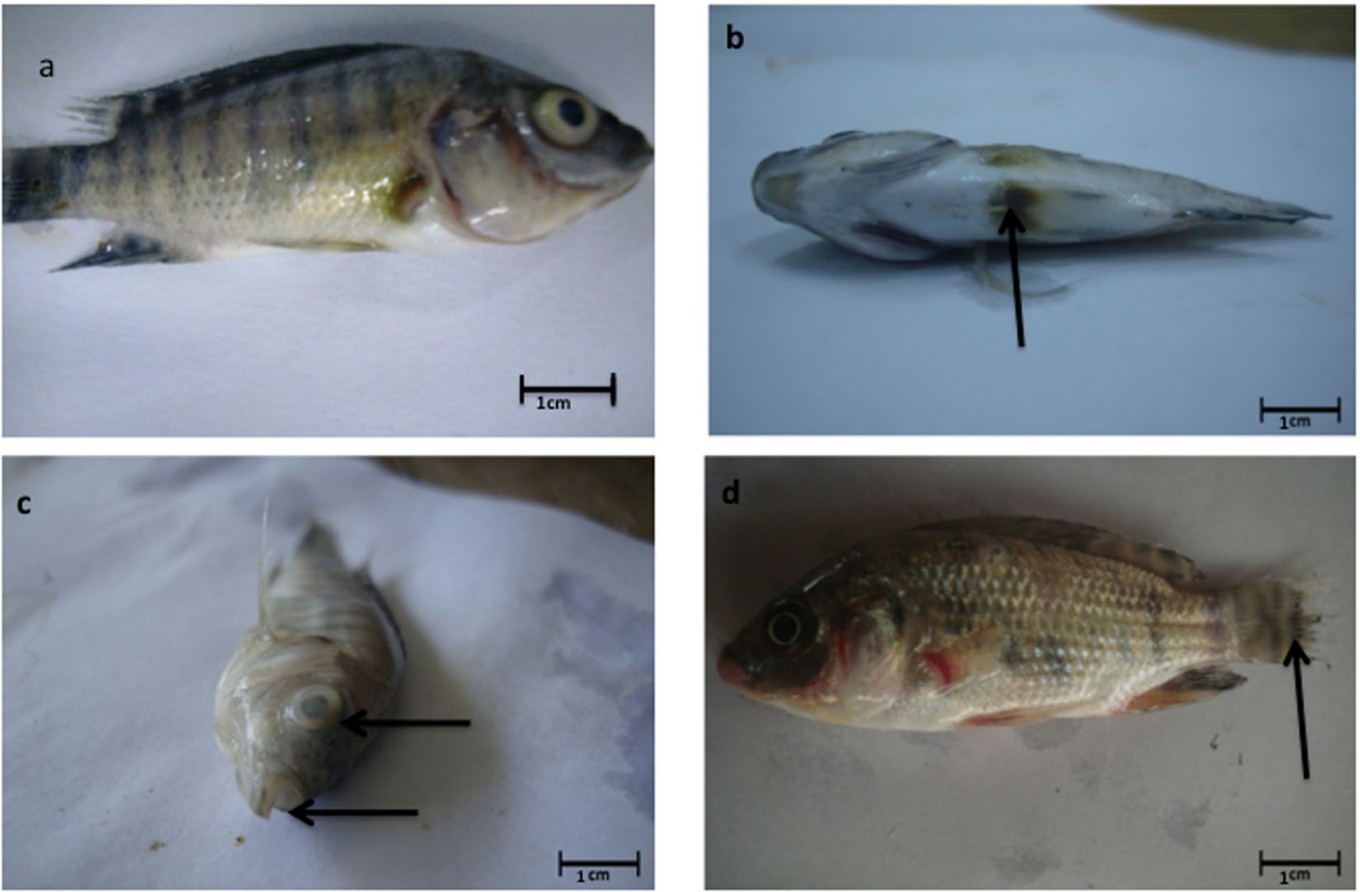
Artificially infected fish with *E*. *faecalis* isolate expressing distinct disease symptoms. (**a**) Control; (**b**) Swollen abdomen and hemorrhages at the base of pelvic fins; (**c**) Bilateral opacity and sign of asphyxiation; (**d**) Erosion in caudal fin.

**Figure 3:**
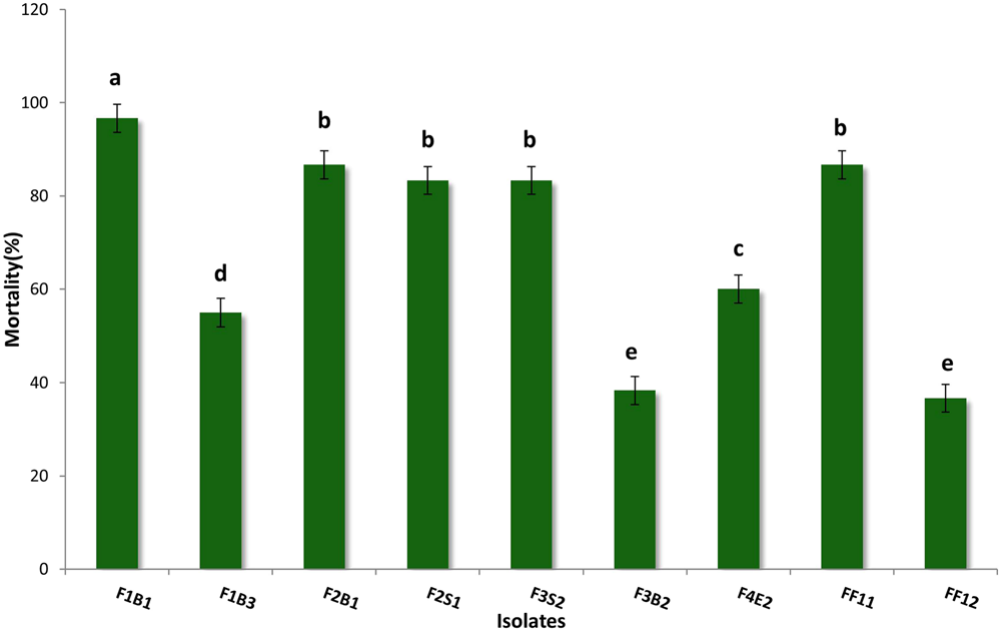
Mortality of tilapia fish (*Oreochromis niloticus*) exposed to fish pathogenic *E*. *faecalis* isolates in the laboratory conditions. One way ANOVA was performed for analyzing the data of three replicated experiment and data in column varies significantly in LSD at p ≤ 0.05. Different letter bars indicates significant variations in mortality of fish in different groups by the *E*. *faecalis* isolates at p ≤ 0.05.

To observe whether *E*. *faecalis* isolates were pathogenic to fish, we conducted an artificial challenge study under laboratory conditions. All 10 tested *E*. *faecalis* isolates produced disease symptoms in artificially infected tilapia (*O*. *niloticus*) except for isolate F4S2. In *O*. *niloticus*, the first clinical sign appeared within 24 hours followed by the death of fish within 72 hours. Major clinical symptoms observed were corneal opacity combined with uni- or bi-lateral exophthalmia, erosion in tail followed by hemorrhage under pelvic fin and signs of asphyxiation (Fig. 2). Among the *E*. *faecalis* isolates, five (F1B1, F2B1, F2S1, F3S2 and FF11), two (F1B3 and F4E2), two (F3B2 and FF12) and one isolate (F4S2) were high, moderate, weak and avirulent, respectively (Fig. 3). We recovered the same bacterial isolates from the abdomen, brain, and tail of artificially challenged fish, and identified them as *E*. *faecalis* based on their phenotypic characteristics.

### Antibiotic Susceptibility Profile

**Figure 4:**
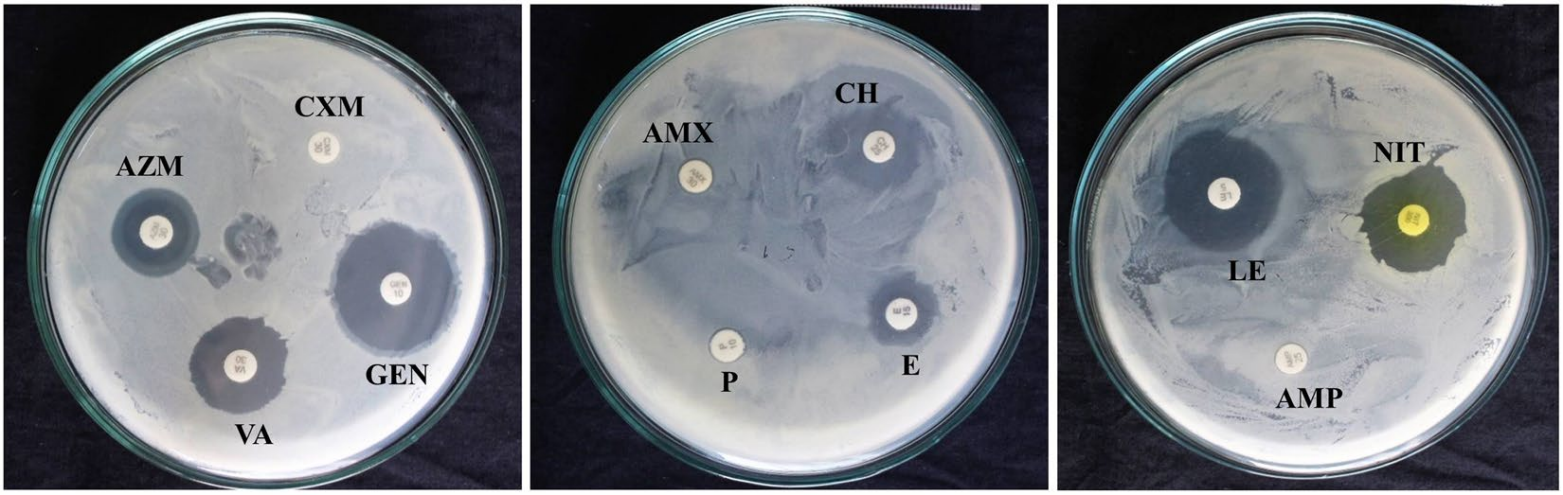
Antibiotic susceptibility profile of fish pathogenic *E. faecalis* FF11 against commercially available antibiotics.

To find out whether the fish pathogenic *E*. *faecalis* isolates had resistance against commercial antibiotics, we screened them against 11 antibiotics using disk diffusion assay. Surprisingly, all of the *E*. *faecalis* isolates displayed resistance to multiple antibiotics *viz*., amoxycillin, ampicillin, cefradine, cefuroxime, erythromycin and penicillin-G (Table 2). However, these isolates exhibited varying levels of susceptibility to nitrofurantoin, azithromycin, gentamycin, levofloxacin, and vancomycin (Fig. 4).

**Table 2:** Antibiotic susceptibility profile of fish pathogenic *E*. *faecalis* isolates from infected fishes in Bangladesh.

Inhibition zone ratio for tested antibiotics											
Isolates	Amoxycillin (AMX)	Ampicillin (AMP)	Azithromycin (AZM)	Cefradine (CH)	Cefuroxime (CXM)	Erythromycin (E)	Gentamicin (GEN)	Levofloxacin (LE)	Nitrofurantoin (NIT)	Penicillin-G (P)	Vancomycin (VA)
F1B1	R	R	2.3 ± 0.0	2.2 ± 0.1	R	R	3.3 ± 0.1	3.4 ± 0.0	3.1 ± 0.1	R	2.2 ± 0.1
F1B3	R	R	2.3 ± 0.1	2.2 ± 0.1	R	R	3.3 ± 0.0	3.4 ± 0.1	3.1 ± 0.0	R	2.1 ± 0.0
F2B1	R	R	2.5 ± 0.2	2.1 ± 0.1	R	R	3.2 ± 0.0	3.5 ± 0.0	3.1 ± 0.1	R	2.3 ± 0.1
F2S1	R	R	2.7 ± 0.2	2.2 ± 0.1	R	R	3.1 ± 0.0	3.5 ± 0.1	3.2 ± 0.1	R	2.2 ± 0.1
F3S2	R	R	2.3 ± 0.1	2.1 ± 0.1	R	R	3.4 ± 0.1	3.4 ± 0.1	3.2 ± 0.1	R	2.1 ± 0.0
F3B2	R	R	2.2 ± 0.1	2.4 ± 0.0	R	R	3.3 ± 0.1	3.2 ± 0.1	3.0 ± 0.0	R	2.2 ± 0.1
F4S2	R	R	2.3 ± 0.0	2.2 ± 0.1	R	R	3.2 ± 0.0	3.2 ± 0.1	3.1 ± 0.0	R	2.2 ± 0.1
F4E2	R	R	2.0 ± 0.0	2.2 ± 0.1	R	R	3.1 ± 0.1	3.5 ± 0.0	3.0 ± 0.0	R	2.1 ± 0.0
FF11	R	R	2.2 ± 0.1	2.2 ± 0.1	R	R	3.3 ± 0.1	3.5 ± 0.1	3.2 ± 0.0	R	2.3 ± 0.0
FF12	R	R	2.2 ± 0.1	2.0 ± 0.0	R	R	3.2 ± 0.1	3.2 ± 0.0	3.2 ± 0.1	R	2.4 ± 0.0

Erythromycin (15 µg disk^−1^), Penicillin (10 µg disk^−1^), Amoxycillin (30 µg disk^−1^), Vancomycin (30 µg disk^−1^), Ampicillin (25 µg disk^−1^), Levofloxacin (5 µg disk^−1^), Cefuroxime (30 µg disk^−1^), Azithromycin (30 µg disk^−1^), Nitrofurantoin (30 µg disk^−1^), Cefradine (25 µg disk^−1^), Gentamicin (10 µg disk^−1^), R = Resistant. Disk diameter is 6.0 mm. Data are presented as Mean ± SE (n = 3).

### *In Vitro* Inhibitory Effects of Plant Extracts

To see whether plant extracts can inhibit the growth of fish pathogenic *E*. *faecalis* isolates, both aqueous and polar extracts of 23 medicinal plants were screened *in vitro*. Out of the 23 aqueous extracts tested, the extracts of *Tamarindus indica* and *Emblica officinalis* leaves, *Allium sativum* bulb, and *Syzygium aromaticum* buds remarkably inhibited the growth of *E*. *faecalis* (Table 3). Among the active extracts, *T*. *indica* and *E*. *officinalis* exhibited low to moderate antibacterial activity with bacteriostatic effects against all *E*. *faecalis* isolates tested. *A*. *sativum* inhibited the bacterial growth with high zones of inhibition with a bacteriostatic effect. The crude aqueous extracts of *S*. *aromaticum* resulted moderate zone of inhibition in disk diffusion assay with bactericidal activity against all *E*. *faecalis* isolates.

**Table 3:** *In vitro* inhibitory activities of disk containing aqueous extracts of medicinal plants of Bangladesh against fish pathogenic *E*. *faecalis* isolates.

Name of plant species	Type of inhibition	Inhibition zone ratio for tested aqueous extracts of medicinal plant										
		F1B1	F1B3	F2B1	F2S1	F3S2	F3B2	F4S2	F4E2	FF11	FF12	Avg. zone
*Tamarindus indica*	Bacteriostatic	1.8 ± 0.1	1.7 ± 0.2	1.8 ± 0.1	1.8 ± 0.1	1.6 ± 0.2	1.8 ± 0.1	1.8 ± 0.2	1.6 ± 0.1	1.8 ± 0.0	1.7 ± 0.2	1.7 ± 0.02
*Emblica officinalis*	Bacteriostatic	1.2 ± 0.0	1.2 ± 0.1	1.3 ± 0.0	1.1 ± 0.1	1.2 ± 0.1	1.1 ± 0.2	1.2 ± 0.6	1.3 ± 0.1	1.3 ± 0.2	1.4 ± 0.2	1.2 ± 0.03
*Allium sativum*	Bacteriostatic	3.0 ± 0.2	2.7 ± 0.2	3.1 ± 0.3	2.9 ± 0.3	2.7 ± 0.0	2.9 ± 0.3	2.8 ± 0.1	3.0 ± 0.1	2.7 ± 0.3	2.8 ± 0.2	2.9 ± 0.04
*Syzygium aromaticum*	Bactericidal	2.6 ± 0.2	2.4 ± 0.1	2.6 ± 0.1	2.6 ± 0.1	2.4 ± 0.1	2.6 ± 0.1	2.6 ± 0.2	2.6 ± 0.0	2.3 ± 0.1	2.5 ± 0.1	2.5 ± 0.04

8 mm filter paper disc soaked with aqueous extracts of *T*. *indica*, *E*. *officinalis*, *A*. *sativum* and *S*. *aromaticum* (30 µl disk^−1^) were used. Data are presented as Mean ± SE (n = 3).

**Figure 5:**
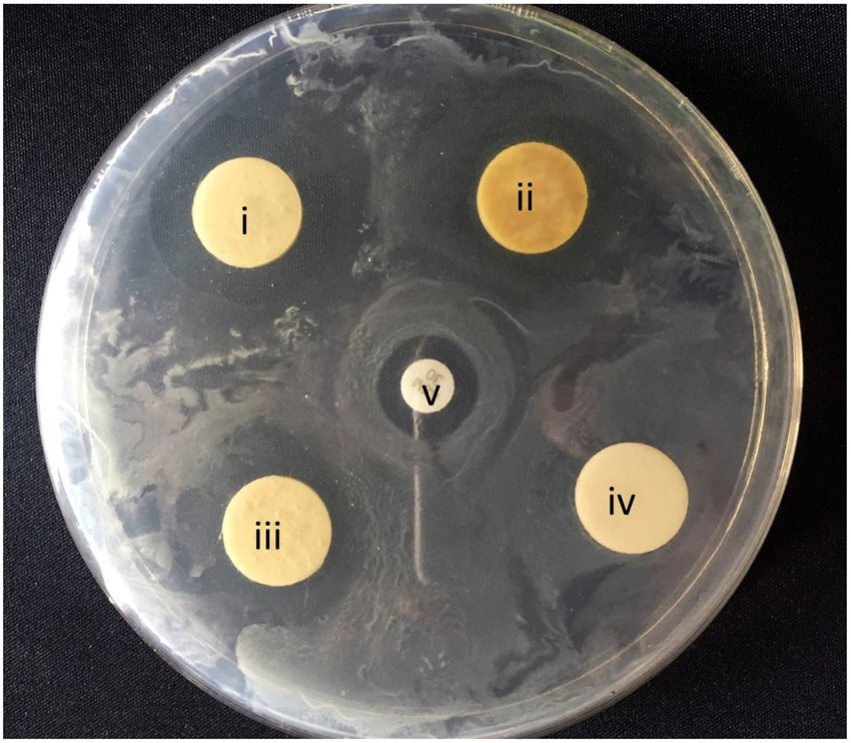
*In vitro* antibacterial activity of disk containing solvent extract of *Allium sativum* and *Syzygium aromaticum*. Note: (i) methanol extract of *S.* *aromaticum﻿*, (ii) acetone extract of *S*. *aromaticum*, (iii) Acetone extract of *A*. *sativum*, (iv) Blank control, (v) Azithromycin antibiotic disk.

In case of the organic extracts of medicinal plants, the methanol extracts of *A*. *sativum* exhibited antibacterial activity against the *E*. *faecalis* isolates (Table 4). On the other hand, moderate and moderate to high zone of inhibition with bactericidal effects were recorded in *n*-hexane and methanol and acetone extracts of *S*. *aromaticum*, respectively (Fig. 5).

**Table 4:** *In vitro* inhibitory activities of disk containing different organic extracts of *Syzygium aromaticum* and *Allium sativum* against fish pathogenic *E*. *faecalis* isolates.

Treatment		Inhibition zone ratio for tested organic extracts of medicinal plants									
Plant Extract		F1B1	F1B3	F2B1	F2S1	F3B2	F3S2	F4E2	FF11	FF12	Avg. zone
*A*. *sativum*	Methanol extract^*^	2.1 ± 0.0	2.2 ± 0.2	1.9 ± 0.0	1.9 ± 0.1	1.8 ± 0.2	2.2 ± 0.2	2.1 ± 0.0	2.2 ± 0.1	1.9 ± 0.2	2.0 ± 0.05
*S*. *aromaticum*	Methanol extract^*^	2.0 ± 0.1	2.1 ± 0.2	2.3 ± 0.0	2.2 ± 0.1	2.0 ± 0.0	2.1 ± 0.2	2.2 ± 0.1	2.4 ± 0.1	2.1 ± 0.1	2.2 ± 0.04
	Acetone extract^*^	2.0 ± 0.1	2.0 ± 0.1	2.1 ± 0.2	2.1 ± 0.1	2.2 ± 0.2	2.0 ± 0.1	1.9 ± 0.0	2.1 ± 0.0	2.1 ± 0.2	2.1 ± 0.03
	*n*-Hexane extract^*^	1.4 ± 0.1	1.3 ± 0.1	1.4 ± 0.1	1.4 ± 0.0	1.2 ± 0.1	1.2 ± 0.0	1.4 ± 0.0	1.4 ± 0.1	1.2 ± 0.1	1.3 ± 0.03

8 mm filter paper disk soaked with methanol extract of *A*. *sativum*, methanol extract of *S*. *aromaticum*, acetone extract of *S*. *aromaticum* and *n*-hexane extract of *S*. *aromaticum* (30 µl disk^−1^) were used. Data are presented as Mean ± SE (n = 3).

### Determination of Minimum Inhibitory Concentration for the Effective Extracts

For determining minimum inhibitory concentration (MIC) of the organic extracts of *A*. *sativum* and *S*. *aromaticum*, a quantitative bioassay was carried out against a highly virulent strain of *E*. *faecalis* FF11 using disk diffusion method. Bioassay revealed that the MIC against the most highly virulent pathogen for methanol extract of *A*. *sativum* was 62.5 µg ml^−1^. On the other hand, MICs for a methanol and acetone extracts of *S*. *aromaticum* were 62.5 µg ml^−1^, and *n*-hexane extract of *S*. *aromaticum* was 125 µg ml^−1^.

### *In Vivo* Effects of Plant Extracts as Preventive Agents

To know whether the medicinal plant extracts are effective in prevention of disease caused by *E*. *faecalis*, an *in vivo* bioassay was carried out. The fish were fed with various extracts before exposing them to the highly virulent isolates of *E*. *faecalis*. As there were variation in the untreated control survival rate, so the data was analyzed using relative percentage of survival (RPS) method comparing the percentage of fish that survived in the treatment group against the control group without treatment. Moderate to high (66.7 to 75.0%) relative percentage of survival (RPS) of fish was recorded when a methanol extract of *A*. *sativum* or acetone and methanol extracts of *S*. *aromaticum* were applied as preventive agents against *E*. *faecalis* infection (Table 5). Although prevention of disease by plant extracts varied against different isolates of *E*. *faecalis*, the average relative percentage of survival of fish against high, moderate and weak virulent isolates for any of the plant extracts were very similar to each other.

**Table 5:** Relative percentage of survival (RPS) of fish fed with medicinal plant extracts as preventive agents in artificial infection challenge with fish pathogenic *E*. *faecalis*.

Treatment	RPS (%)											
	High Virulent Strains						Moderate Virulent Strains			Low Virulent Strains		
	F1B1	F2B1	FF11	F2S1	F3S2	Avg. RPS	F1B3	F4E2	Avg. RPS	F3B2	FF12	Avg. RPS
*A*. *sativum* Methanol extract	80	70	70	75	75	74	67	75	71	100	50	75
*S*. *aromaticum* Acetone extract	60	80	80	100	50	74	75	75	75	100	50	75
*S*. *aromaticum* Methanol extract	40	60	80	100	75	71	67	67	67	75	75	75
*S*. *aromaticum n*-Hexane extract	60	60	80	50	50	65	50	67	58	50	50	50

### *In Vivo* Effects of Plant Extracts as Therapeutic Agents

**Figure 6:**
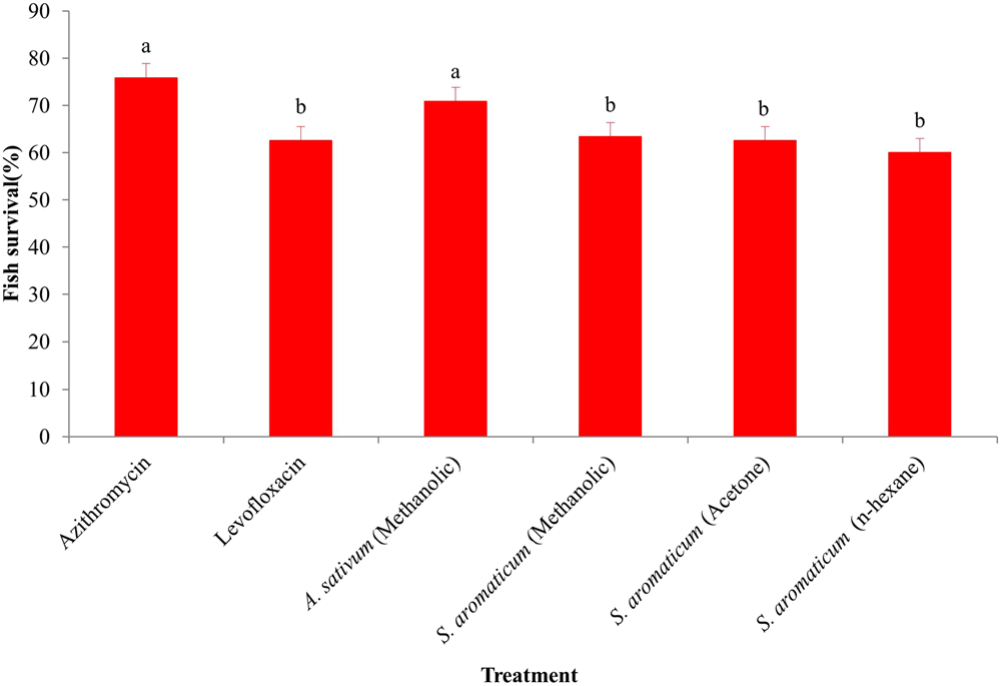
Average survival rate (therapeutic effects) of *O*. *niloticus* fed with medicinal plant extracts and commercial antibiotics after infection challenge by a high virulent strain of *E*. *faecalis*. One way ANOVA was performed for analysis of the data and mean values in the bars followed by the same letter(s) are not significantly different as assessed by LSD (least significance difference) at p ≤ 0.05.

Effects of organic extracts of *A*. *sativum* and *S*. *aromaticum* as therapeutic agents were evaluated in laboratory conditions that compared them with two commercial antibiotics, azithromycin and levofloxacin. The fish treated with azithromycin and levofloxacin exhibited average survival of 75.8 ± 1.5% and 62.5 ± 1.2%, respectively. On the other hand, survival of fish treated with methanol extracts of *A*. *sativum* was 70.8 ± 4.2% (Fig. 6), which did not vary significantly compared to treatment with azithromycin. A moderate survival rate (60.0 ± 2.2 to 63.3 ± 1.6%) was recorded in fish treated with acetone, methanol or *n*-hexane extracts of *S*. *aromaticum*. No survival of fish in the untreated control group was found.

## Discussion

*Enterococcus* sp. has frequently been isolated from various disease-infected fishes in Bangladesh^10, 11^. We identified and characterized ten virulent strains of *E*. *faecalis* isolated from infected tilapia and catfish collected from local fish farms in Bangladesh. Our 16S rRNA gene sequencing data, PCR study with specific primer, and artificial challenge study confirmed that all of these 10 isolates are *E*. *faecalis*. Furthermore, we found that all of these *E*. *faecalis* were resistant to several antibiotics but highly susceptible to the crude extracts of two medicinal herbs, *A*. *sativum* and *S*. *aromaticum*. Methanol extracts of *A*. *sativum* and methanol and acetone extracts of *S*. *aromaticum*, when used as both therapeutic and preventive significantly reduced the mortality of tilapia fish artificially infected with *E*. *faecalis*. This study for the first time confirmed and identified *E*. *faecalis* as a virulent pathogen causing high mortality in tilapia and catfish in Bangladesh.

*E. faecalis* strains have been reported as antibiotic resistant bacteria in many countries^14^. In the present study, all of the *E*. *faecalis* isolates showed resistance to multiple antibiotics. Enterococci are able to acquire transposons, resistance plasmids, and sex pheromone plasmids from a wide range of recipients, which facilitate them to act as a reservoir of resistance genes^17^. Resistance of Enterococci to various antibiotics such as chloramphenicol, clindamycin, erythromycin, tetracycline, aminoglycosides, beta-lactamases and vancomycin has been reported^18, 19^. Various antibiotics and growth promoting agents are widely used in aquaculture, livestock and poultry rearing facilities in Bangladesh without proper awareness about their application. This may lead to the resistance of the fish pathogen *E*. *faecalis* to multiple antibiotics shown in this study. Resistance of *E*. *faecalis* to various cell wall degrading antibiotics such as penicillin-G, ampicillin and vancomycin has been reported^19–21^. In this study, all *E*. *faecalis* isolates were found to be resistant to penicillin-G and ampicillin but, sensitive to vancomycin, which is very rare for this pathogen. The antibiotic susceptibility profile of a fish pathogenic *E*. *faecalis* demonstrated in this article has not previously been reported.

One of the interesting findings of this study is that multiple antibiotic resistant strains of *E*. *faecalis* show high susceptibility to both aqueous and organic extracts of several herbal medicines including *A*. *sativum*, and *S*. *aromaticum* (Table 4). Both methanol and acetone extracts of these medicinal plants displayed higher inhibitory activity against *E*. *faecalis* compared to ethyl acetate and *n*-hexane extracts, indicating that the active compounds are relatively polar secondary metabolites. This hypothesis is supported by the inhibitory effects of aqueous extracts of the medicinal plants against the *E*. *faecalis in vitro*. A further bioassay-guided chromatographic fractionation and purification process would lead to the discovery of bioactive secondary metabolites from *A*. *sativum*, and *S*. *aromaticum* extracts against the multiple antibiotic resistant *E*. *faecalis*.

Another noticeable finding of this study is that organic extracts of *A*. *sativum* and *S*. *aromaticum* significantly increase the survival rate of tilapia from infection by *E*. *faecalis* in artificial infection. (Table 5). We also demonstrated that these plant extracts were equally effective as a therapeutic agent against the disease caused by *E*. *faecalis*. The methanol extract of *A*. *sativum* provided significant recovery of fish against the infection by the most virulent strains of *E*. *faecalis*, which exhibited equivalent efficacy to azithromycin. Moderate rates of survival were also obtained in this study in fish treated with acetone, methanol or *n*-hexane extracts of *S*. *aromaticum* against *E*. *faecalis* infection that were similar to the treatment of fish with levofloxacin. In this study, organic extracts of *A*. *sativum* and *S*. *aromaticum* provided significant prevention and survival of fish against *E*. *faecalis* infection. Several antibacterial secondary metabolites have been discovered from both *A*. *sativum* and *S*. *aromaticum* ^22^. Among the bioactive compounds, ajone and allicin present in *A*. *sativum*, are commonly known to be active against Gram positive pathogenic bacteria. The antibacterial effects of clove (*S*. *aromaticum*) essential oils have been reported^23–27^. This report for the first time demonstrated both preventive and therapeutic efficacies of the organic extracts of *A*. *sativum* and *S*. *aromaticum* against *E*. *faecalis* infection in tilapia. These findings indicate that medicinal plant extracts could be used as a natural alternative to the synthetic antibiotics to control enterococcal infection in fish. As crude extracts contains multiple secondary metabolites, chances of the development of resistance against crude plant extracts are likely to be lesser than those of pure antibiotics.

In summary, we isolated 10 strains of multiple antibiotic resistant *E*. *faecalis* with varying levels of virulence from naturally infected tilapia and catfish and identified them through phenotypic properties, 16S rRNA gene sequencing and specific PCR primers. Although the *E*. *faecalis* isolates demonstrated resistance to multiple antibiotics, they showed remarkable susceptibility to both aqueous and organic extracts of *A*. *sativum* and *S*. *aromaticum*. Application of methanol and acetone extracts of these herbal medicines through feed effectively prevented and suppressed infection in tilapia fish challenged with virulent strains of *E*. *faecalis*. Taken together, our results suggest that medicinal plant extracts could be used as a potential alternative to the synthetic antibiotics to control fish diseases cause by *E*. *faecalis* in aquaculture. A further study is required to elucidate the mode of action of the plant crude extracts in preventing and/or recovering fish disease caused by *E*. *faecalis*.

## Methods

### Collection of Fish Sample

A number of 17 tilapia (*Oreochromis niloticus*) and 8 catfish (*Clarias batrachus* and *Heteropneustes fossilis*) suspected of being infected with *Enterococcus* sp. were collected from several fish farms located at the Gazipur district of Bangladesh. The external symptoms observed in infected fish samples were excess secretion of slime, protruding opaque eyes, and swollen abdomen. Hemorrhages under the pelvic fin region and erosion in the tail were also observed in the infected fish. Decrease in appetite, lethargy, erratic swimming, and spinning movements before death were also noticed in infected and moribund fish.

### Isolation of Pathogen and Phenotypic Identification

Bacteria were isolated from skin surface, tail, gut, eye and brain of the infected fish on KF streptococcal agar media (Himedia, India). The isolates were subcultured on nutrient agar (Micromaster, India) and maintained in nutrient broth (Micromaster, India) media. Preliminary phenotypic identification of the isolates was performed following the standard morphological, physiological and biochemical analyses^28^.

### Molecular Identification of Bacterial Isolates

Ten randomly selected representative isolates were used for molecular identification through 16S rRNA gene sequencing. They were cultured in 10 ml test tubes containing nutrient broth at 28 °C and 120 rpm in a shaking incubator for 24 hours. Genomic DNA of the isolates was extracted by using sodium acetate for PCR analysis followed by sequencing. Briefly, bacterial cells were harvested up to 2 × 10^6^ cell ml^−1^ into 1.5 ml microcentrifuge tubes for centrifugation for 10 min at 5000 × g. One milliliter of 95% ethanol was added to the harvested bacterial cells followed by the addition of 50 µl of 3 M sodium acetate before incubating the tube at −20 °C for 2 hours. The sample was centrifuged for 2 minutes at 13000 × g and the supernatant was discarded. One milliliter of 100% ethanol was added to the tube and incubated at 20 °C for 1 minute. The ethanol was removed after centrifugation at high speed and the precipitated DNA was allowed to dry for 5 minutes and then rehydrated in 50 µl of 1 × TE buffer. The extracted DNA was either freshly used for PCR or stored at −20 °C for further use. The PCR reaction mixture was prepared following standard protocols^29^. The 16S rRNA gene from the genomic DNA was amplified with universal primer set (27F and 1492R) in a PCR thermocycler (Eppendrof Master Cycler). The PCR amplification was done by an initial denaturation at 94 °C for 5 min; 35 cycles of a denaturation at 94 °C for 1 min, an annealing at 57 °C for 40 sec and an extension at 72 °C for 1 min and a final extension step at 72 °C for 10 min.

The PCR amplicons were purified using Gene JET PCR purification kit (Thermo Scientific Inc., USA) following the manufacturer’s purification protocol and sequenced at the Center for Advanced Research in Sciences (CARS) of University of Dhaka in Bangladesh. Analysis of the sequenced data was done using the BLAST program available in National Center for Biotechnology Information (NCBI) website (www.ncbi.nlm.nih.gov). The identified isolates were stored in freezer in 10% glycerol.

### *E*. *faecalis* Specific Primer Based PCR

In order to develop a PCR protocol for specific detection of *E*. *faecalis*, sequences of the 16S rRNA genes of the ten *E*. *faecalis* isolates and sequences available in the NCBI GenBank database were aligned using the CLUSTAL W software program and forward and reverse primers were designed from the conserved regions of *E*. *faecalis* 16S rDNA sequences based on the alignment. In addition, the universal reverse primer 1492R was also used in this study. Sequences of the designed primers are given in Table 6. The theoretical specificity of each primer was determined by matching the primers to the Ribosomal Database Project II (RDP-II) using the CHECK-PROBE function^30^. The designed primers were purchased from Sigma Ltd.

**Table 6:** Sequences, size and GC contents of primers used for PCR amplification.

Primers	Sequence (5′-3′)	Nucleotide numbers in *E*. *coli* 16S rDNA sequence	Primer size (bp)	GC content (%)
EfacF1	CGTTAGTAACTGAACGTC	470–488	18	44.44
Efac R1	GACCGCGAGGTCATGCA	1353–1370	17	64.7
Universal 1492R	GGATACCTTGTTACGACTT	1473–1492	19	42.1

PCR amplification was conducted in a PCR thermocycler (Eppendrof Master Cycler) which included an initial denaturation at 94 °C for 5 min and 35 cycles comprising denaturation at 94 °C for 1 min, annealing at 48 °C for 40 s, and extension at 72 °C for 1 min and a final extension at 72 °C for 10 minutes with a hold temperature of 4 °C. Amplified PCR products were separated by loading 5 μl of PCR products mixed with 1 μl of 6 × gel loading dye (Thermo Scientific Inc., USA) in 1.5% agarose gel and electrophoresis was run at 100 Volt for 40 minutes. DNA ladder (1 Kb DNA ladder, Bioneer Ltd.) was also loaded in the gel to determine size of the PCR amplicons. The amplicons were visualized in a gel documentation system (Kita G-1000, Germany) after ethidium bromide staining.

### *In vivo* Infection Model

Young Nile tilapia (*O*. *niloticus*; average weight 15.0 ± 2.0 g) was collected from rearing pond of the Faculty of Fisheries, Bangabandhu Sheikh Mujibur Rahman Agricultural University, Gazipur, Bangladesh where no previous occurrence of Streptococcal or Enterococcal infection in fish was recorded. The fish were kept in aquarium in the laboratory at room temperature for two weeks, fed twice daily with commercial pellets and observed carefully for any sign of disease or abnormality in their behavior. Prior to challenge study, 10 randomly chosen tilapia fish were sacrificed and their kidneys, eye, skin and fins were aseptically collected and spread on KF Streptococcal agar medium (Himedia, India) and incubated at 28 °C for 72 hours to observe pre-existence or colonization of Enterococcal pathogens in the fish. The experimental fish were first anesthetized by using MS222 and then bathed in bacterial suspension for (2–4 × 10^5^ cfu ml^−1^) for 15 minutes. The fish were transferred to an aquarium containing 20 L of fresh underground water. Microbiological evaluation of the water used in the aquarium was also done before initiation of the experiment. Twenty fish was kept in each aquarium and three replicated aquaria were used for each of the bacterial isolates used in the study. Continuous aeration was maintained in the aquarium. The fish were fed with commercial feed pellets at 5% of their body weight. Fifty percent of the aquarium water was exchanged every day. A control group fish (without immersion in bacterial suspension) was maintained in three aquaria under same condition. Disease incidence in the tilapia fish was recorded at 7 days duration.

### *In vitro* Antibiogram Assay

Susceptibility profile of isolated 10 fish pathogenic *E*. *faecalis* isolates to various commercial antibiotic disks was determined by disk diffusion method^31^. The antibiotic disks used in this study were erythromycin (15 µg disk^−1^), penicillin (10 µg disk^−1^), amoxycillin (30 µg disk^−1^), vancomycin (30 µg disk^−1^), ampicillin (25 µg disk^−1^), levofloxacin (5 µg disk^−1^), cefuroxime (30 µg disk^−1^), azithromycin (30 µg disk^−1^), nitrofurantoin (30 µg disk^−1^), cefradine (25 µg disk^−1^), gentamicin (10 µg disk^−1^). Bacterial culture was spread on the Isosensei Test Agar plates (Micromaster, India), antibiotic disks were aseptically placed on the culture plate and incubated at 37 °C for 24 h in an incubator. After incubation, the diameter of zone of inhibition was measured, and the isolates were termed resistant according to CLSI-specified interpretive criteria^32^.

### Extraction and Inhibitory Activity of Medicinal Plant Extract

The inhibitory activities of crude aqueous extracts of 23 medicinal plants selected based on their medicinal properties^33, 34^ were screened on *E*. *faecalis* isolates. For this purpose, desired parts of plants were rinsed with sterilized distilled water and cut into small pieces. Then the small pieces were weighed and grounded using a mortar and pastel. Sensitivity of the *E*. *faecalis* isolates to crude aqueous plant extracts was determined as described elsewhere^33^. Data were collected for three replicated plates for each of the crude medicinal plant extracts to individual isolates.

To know more details about the active principles in the active plant extracts, the plant extracts were successively fractionated with *n*-hexane, ethyl acetate, acetone, methanol and then bioassay was performed against the pathogenic isolates using the disk diffusion method. For fractionation, fresh plant materials were dried till all aqueous portions are removed. 25 g of dried plant materials were taken from each sample and ground to powder form using electric blender. The powdered plant material was added into 100 ml *n*-hexane, ethyl acetate, acetone, methanol for each sample. They were incubated 72 hours in orbital shaker at room temperature. The extracts were then evaporated in rotary evaporator at 50 °C. The dried extract samples were later dissolved in distilled water making 25 mg ml^−1^ solvent^35^. For performing disk diffusion method, sterilized filter paper disks (8 mm in size) were soaked with 30 µl extracts (25 mg ml^−1^ solvent) and kept overnight at room temperature and dried aseptically to ensure complete evaporation of the solvent. A suspension of fresh culture of experimental bacteria was prepared and 30 µl of bacterial inoculum was spread over the Isosensei Test Agar plate with a sterilized glass rod. Then the disks containing fraction of herbal extracts or untreated as control (without extract) were carefully dispensed at uniform distances over the surface of bacterial culture. All plates were incubated at 28 °C for 24 h. After incubation, plates were observed for formation of inhibitory zone on the microbial lawns. Diameter of the disks and diameter of the zone of inhibitions were measured and ratios between the diameters were calculated^32^. Each treatment was replicated for three times.

### Determination of MIC

To determine the minimum inhibitory concentration (MIC) of four types of organic solvent extracts of medicinal plants, serial two-fold dilution technique was followed. Dilutions were adjusted at 1000 µg ml^−1^, 500 µg ml^−1^, 250 µg ml^−1^, 125 µg ml^−1^, 62.5 µg ml^−1^ and 31.25 µg ml^−1^ (w/v) and the disks were prepared as described earlier. The MIC was determined against a highly virulent strain (FF11) following the disk diffusion method. Three replications were used for each dilution. Thirty microliter of bacterial culture having a concentration of 10^5^ cfu ml^−1^ was inoculated in each culture plate. The growth of bacteria that were decrease in the next dilution was considered as MIC value^32^.

### *In vivo* Effects of Plant Extracts as Preventive Agents

*In vivo* effects of methanol extract of garlic (*A*. *sativum*) and methanol, acetone and *n*-hexane extracts of clove (*S*. *aromaticum*) as preventive agents against *E*. *faecalis* infection on fingerlings of *O*. *niloticus* were determined under laboratory condition. For this purpose, 25 mg ml^−1^ methanol extract of garlic and *n*-hexane, methanol and acetone extract of *S*. *aromaticum* was prepared. These extracts were mixed separately with commercial fish feed by spraying and dried overnight at room temperature. For this purpose, five groups of fish (270 fish in each group) was maintained in five separate aquarium of which one group was fed normal commercial fish feed while the other groups were fed any of the four plant extracts (375 mg kg^−1^ fish). We selected the dose of plant extracts from an earlier study for a highly virulent strain F1B1^12^. Fish were fed the feed at a rate of 5% of their body weight twice a day for 14 days. After that, each group of fish was artificially infected with nine virulent strains of *E*. *faecalis* each of which had three replications (10 fish in each aquarium) following the bath challenge method as described earlier and observed for 7 days. After initiation of artificial infection the fish were fed normal commercial fish feed at the same rate and mortality of the fish in each aquarium was recorded. Continuous aeration and daily exchange of 50% water was maintained throughout the study period. At the end of the experiment the average mortality of fish for each treatment was calculated. Since, virulence level of *E*. *faecalis* isolates varied in the control group relative percent survival (RPS)^36^ and average relative percent survival of fish in each treatment was analyzed.

### *In Vivo* Effects of Plant Extracts as Therapeutic Agents

Therapeutic potentials of methanol extract of garlic (*A*. *sativum*) and methanol, acetone and *n*- hexane extracts of clove (*S*. *aromaticum*) on the *O*. *niloticus* fingerlings against a highly virulent isolate F1B1 was evaluated in *in vivo* condition. Two commercial antibiotics, azithromycin and levofloxacin were also used to compare the property of plant extracts with the commercial antibiotics. The fish (*O*. *niloticus*) were exposed to bacterial suspension of the *E*. *faecalis* isolate F1B1 as described earlier and then seven groups of fish each of which have 3 replications (n = 10) were kept in separate aquarium. Different group of fish were fed commercial fish feed mixed with methanol extract of garlic (375 mg kg^−1^ fish), methanol extract of clove (375 mg kg^−1^ fish), acetone extract of clove (375 mg kg^−1^ fish), *n*-hexane extract of clove (375 mg kg^−1^ fish), azithromycin (10 mg kg^−1^ fish), levofloxacin (14 mg kg^−1^ fish) and normal fish feed (control group). The fish were feed twice a day and the experiment was continued for 7 days. External disease symptoms and abnormal behaviors in the treated fish were observed. Aeration was maintained throughout the experiment. Around 50% water of the aquarium was exchanged in two days interval.

### Statistical Analysis of Data

Experiments for *in vivo* infection model of *E*. *faecalis* isolates and evaluation of biological activities of the crude extracts were carried out using a complete randomized design (CRD). Data were analyzed by one-way analysis of variance (ANOVA) and the mean values were separated by LSD (least significant difference) posthoc statistic. All the analyses were performed using SPSS (IBM SPSS statistics 21, Georgia, USA). Mean value ± standard error of 3 replications was used in Tables and Figures.

### Ethical Statement

The animal experiment in this study were carried out following guidelines and recommendations of “Guidelines for the Use of Fishes in Research” published by American Fisheries Society (2014) since there is no specific guideline for use of fish in research in Bangladesh. However, the research works were strictly supervised by an advisory committee of research works of M. R. with the approval of Dean, Graduate studies, BSMRAU. The advisory committee monitored the research works considering the ethical issues.

## Electronic supplementary material

Table S1 and S2
