# Supplementary material for: Molecular Identification of Multiple Antibiotic Resistant Fish Pathogenic Enterococcus faecalis and their Control by Medicinal Herbs
Source: Sci Rep. 2017 Jun 16;7:3747. doi: 10.1038/s41598-017-03673-1 (PMC5473830; doi:10.1038/s41598-017-03673-1)
Supplement: Supplementary file 1 — Table S1 and S2 [file 41598_2017_3673_MOESM1_ESM.doc]

**Molecular Identification of Multiple Antibiotic Resistant Fish Pathogenic *Enterococcus***

***faecalis* and their Control by Medicinal Herbs**

Muntasir Rahman^1^, Md. Mahbubur Rahman^1*^, Suzan Chandra Deb^1,2^, Md. Shahanoor Alam^3^, Md. Jahangir Alam^4^, and Md. Tofazzal Islam^1*^

^1^Department of Biotechnology, Bangabandhu Sheikh Mujibur Rahman Agricultural University,

Gazipur-1706, Bangladesh

^2^Department of Genetic Engineering and Biotechnology, Shahjalal University of Science and

Technology, Sylhet-3114, Bangladesh

^3^Department of Genetics and Fish Breeding, Bangabandhu Sheikh Mujibur Rahman Agricultural

University, Gazipur- 1706, Bangladesh

^4^Department of Fisheries Biology and Aquatic Environment, Bangabandhu Sheikh Mujibur

Rahman Agricultural University, Gazipur-1706, Bangladesh

*Corresponding Authors

**Table S1.** Source of isolation, genbank accession number and 16S rRNA gene sequence homology of *E. faecalis* isolates with *E. faecalis* strain ATCC 19433

| Isolate No. | Host fish | Organ of Fish | NCBI genbank accession No. | Sequence homology with *E. faecalis* strain ATCC 19433 |
| --- | --- | --- | --- | --- |
| F1B1 | *Heteropneustes fossilis* | Brain | KX065363 | 99.93 |
| F1B3 | *H. fossilis* | Brain | KY486240 | 99.72 |
| F2B1 | *Oreochromis niloticus* | Brain | KY486242 | 99.87 |
| F2S1 | *O. niloticus* | Skin | KY486246 | 99.63 |
| F3S2 | *Heteropneustes fossilis* | Skin | KY486244 | 99.72 |
| F3B2 | *H. fossilis* | Brain | KY486241 | 99.72 |
| F4S2 | *O. niloticus* | Skin | KY486245 | 99.86 |
| F4E2 | *O. niloticus* | Eye | KY486243 | 99.86 |
| FF11 | *O. niloticus* | Gut | KX065364 | 99.93 |
| FF12 | *Clarias gariepinus* | Gut | KY486247 | 99.62 |

**Table S2.** List of medicinal plants used to observe inhibitory activity against fish pathogenic *E. faecalis*

| **SL**  **No.** | **Local Name** | **English Name** | **Scientific Name** | **Plant Parts used** |
| --- | --- | --- | --- | --- |
| 1 | Ada | Zinger | *Zingiber officinale* | Rhizome |
| 2 | Holud | Turmeric | *Curcuma longa* | Rhizome |
| 3 | Labango | Clove | *Syzygium aromaticum* | Flower Bud |
| 4 | Rosun | Garlic | *Allium sativum* | Bulb |
| 5 | Peyaj | Onion | *Allium cepa* | Bulb |
| 6 | Kaligira | Black cumin | *Nigella Sativa* | Seed |
| 7 | Mehogoni | Leaved Mehogoni | *Azadirachta indica* | Seed |
| 8 | Lau | Bottle gourd | *Laganaria siceraria* | Seed, Fruit |
| 9 | Morich | Dry chilli | *Capsicum pendulum* | Fruit |
| 10 | Peyara | Guava | *Psidium guajava* | Fruit |
| 11 | Jolpai | Olive | *Olea europaea* | Fruit |
| 12 | Kachamorich | Chilli | *Capsicum pendulum* | Fruit |
| 13 | Noyontara | Rose periwinkle | *Catharanthus roseus* | Leaf, Flower |
| 14 | Amloki | Amla | *Emblica officinalis* | Leaf |
| 15 | Tatul | Tamarind | *Tamarindus indica* | Leaf |
| 16 | Garman lota | Asparagus | *Asparagus officinalis* | Leaf |
| 17 | Arjun | Arjun | *Terminalia arjuna* | Leaf |
| 18 | Papay | Papaya | *Carica papaya* | Leaf |
| 19 | Koromcha | Carunda | *Carissa carandas* | Leaf |
| 20 | Durbaghas | Bermuda grass | *Cynodon dactylon* | Leaf |
| 21 | Neem | Neem | *Azadiracha indica* | Leaf |
| 22 | Dalim | Pomegranate | *Punica granatum* | Leaf |
| 23 | Kamragga | Carambola | *Averrhoa carambola* | Leaf |
